# Safety, probiotic properties, antimicrobial activity, and technological performance of Lactobacillus strains isolated from Iranian raw milk cheeses

**DOI:** 10.1002/fsn3.2365

**Published:** 2021-06-14

**Authors:** Hassan Barzegar, Behrooz Alizadeh Behbahani, Fereshteh Falah

**Affiliations:** ^1^ Department of Food Science and Technology Faculty of Animal Science and Food Technology Agricultural Sciences and Natural Resources University of Khuzestan Mollasani Iran; ^2^ Department of Food Science and Technology Faculty of Agriculture Ferdowsi University of Mashhad Mashhad Iran

**Keywords:** adjunct cultures, Caco‐2 cells, probiotic, proteolytic activity

## Abstract

The objective of this study was to investigate probiotic, antimicrobial, technological and safety properties of lactobacillus strains isolated from local Iranian cheese made from raw milk. Six different samples were prepared, after serial dilution, culture was performed on MRS culture medium. The gram‐positive and catalase‐negative lactobacillus strains were subjected to grouping and identifying using biochemical tests, carbohydrates fermentation profiles, and 16S rDNA analysis. The results of sequence analysis showed the Lactobacillus spp. belonged to *Lactobacillus brevis*, *Lactobacillus acidophilus*, *Lactobacillus plantarum,* and *Lactobacillus casei*. After 3 hr incubation at pH=2, 3–6 log units of strains decreased which *Lactobacillus acidophilus* (B14) and *Lactobacillus brevis* (B2) showed highest resistance to low pH as well as simulated GIT juices. The highest and lowest hydrophobicity degree was belonged to *L. acidophilus* (B14) (65.9%) and *L. casei* (B22) (25.6%), respectively. Also, the highest auto‐aggregation and coaggregation were observed in *L. acidophilus* (B14) (51.3%) and *L*. *plantarum* (B20) (43.6%). The adhered percentage of strains varied from 2.5% to 14.6%. *L. plantarum* (B20) showed highest proteolytic activity followed by *L. acidophilus* (B14). Also, the highest autolytic activity belonged to *L. acidophilus* (B14). All of the strains showed low acidifying potential, except for *L*. *acidophilus* (B17) which decreased 2.05 unit of pH after 24 hr. The isolates did not show lipolytic activity as well as biogenic amines production (except *L. brevis* B3). All of the strains were sensitive to chloramphenicol and erythromycin except *L. acidophilus* (B15) and *L. casei* (B22). All strains showed no hemolysis activity which make them safe for consumption. Based on the obtained results, *L*. *acidophilus* (B14) presented the best probiotic and technological characteristics and is proposed for using as coculture in the dairy industrial.

## Graphical abstract

This study investigates the properties of the isolates especially Lactobacillus acidophilus (B14) when used as probiotics or adjunct culture in dairy food products.

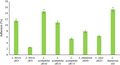

## 1 INTRODUCTION

Functional foods and beverages obtained through fermentation processes, cover major part of the human diet and claimed to have positive effects on health beyond basic nutrition. Fermentation has been used since time immemorial with primary goal of increasing the shelf life of the raw materials, but it also has contributed to the nutritional quality and sensory characteristics of the final product (Bartkiene et al., 2019). The most important bacteria involved in the fermentation process are lactic acid bacteria (LAB). LAB are the most important bacterial groups used to process a variety of dairy products, meat, vegetables, and grains. These bacteria can metabolize the constituents of the food matrix and produce compounds such as peptides, amino acids, aldehydes, alcohols, organic acids, esters, and fatty acids. These compounds play an important role in determining the shelf life, aroma, taste, and texture of fermented foods (Alizadeh Behbahani et al.,^2019^, ^2020^; Mohammed et al., 2018; Saboktakin‐Rizi et al., 2021; Sun et al., 2020). Therefore, LAB are often used as starters in food production processes. Results of clinical studies have confirmed the positive effect of probiotic bacteria on intestinal infections, allergic diseases, improvement of various types of cancer especially clone cancer, improvement of digestion and regulation of the immune system (Ashaolu, 2020; Pratap et al., 2020; Shu et al., 2020; Vasiee et al., 2019; Vasiee, Falah, Sankian, et al., 2020).

In many countries, however, fermented products based on milk, cereals, and other subcultures are known to be beneficial compounds for improving health but many of them have not been studied yet and the health claims have not been adequately supported by credible scientific evidence (Sivamaruthi et al., 2019). Considering a microorganism as a probiotic strain requires in vitro tests following by in vivo investigation. According to the rules of the World Health Organization (WHO), the initial evaluation of the probiotic properties of each microorganism is mandatory by laboratory work. The presence of some probiotic properties is necessary but the presence of others is preferred. The choice of probiotic strains is based on the historical background of their use in food without side effects. The characteristics considered for a strain as a probiotic include bacterial survival during the preparation process, survival in the gastrointestinal tract, ability to attach to intestinal epithelial cells as well as the ability to kill the pathogens (Falah et al., 2019; Vasiee, Falah, Behbahani, et al. 2020).

Investigating the technological properties of LAB can be useful in their industrial applications. In general, the technological potential includes properties that are essential for the bacterial survival and production of compounds that affect the product properties. The important technological features of LAB include autolytic, lipolysis, proteolysis activity, and acid production, which play an effective role in creating the desired flavor in dairy products. Evaluation of the safety of LAB is considered an important feature for the food and feed consumption. Examining the virulence factors of these bacteria is even more important for genera such as Enterococci, which also have the potential for pathogenicity (Mazzola et al., 2017; de Souza & Dias, 2017).

Cheese is a concentrated product of the nutrient components of milk that play an important role in the human diet. Cheese is one of the most widely used dairy products as it is used every day and by people of different ages all over the world. Cheese has a high nutritional value and large amounts of digestible proteins because some proteins are broken down into peptides and amino acids during the process (Ruiz‐Moyano et al., 2019; Tribst et al., 2020). In production of traditional cheeses, the presence of various nonstarter LAB has been observed. It is important to isolate and identify them as well as to study their different characteristics (Patrignani et al., 2019; Plessas et al., 2017). The aim of this study was to investigate the probiotic and technological characteristics of Lactobacillus bacteria isolated from local cheese in Ahvaz, Iran.

## 2 MATERIAL AND METHODS

### 2.1 Isolation and identification of Lactobacillus strains

In order to prevent secondary contamination or changes in the primary microbiota, 6 different samples of local cheese were prepared under hygienic conditions and low temperature. To isolate lactobacilli strains, 5 g of each sample was mixed with 45 ml of sodium citrate (Sigma‐Aldrich, Austria) and homogenization was performed using a stomacher (Interscience, France). Serial dilutions up to 10^–7^ were prepared and culture was done on Man Rogosa and Sharpe agar (MRS agar) (Merck, Germany). The plates were incubated at 37℃ for 48 hr under anaerobic condition (Gas‐pack system). The desired strains which differ in the size and color were isolated and Gram‐staining, catalase testing, biochemical tests, and carbohydrate profiles were performed. Afterward, the genomic DNA was isolated from overnight bacterial culture of each represented strains from desired groups. PCR was done with universal primers, 27FYM, and 1492R based on conserved region of 16S rRNA gene. The reaction was performed in a 0.2 ml microtube with final volume of 20 μl. The polymerase chain reaction (PCR) conditions were as follows: initial denaturation at 94℃ for 8 min and 30 cycles, including 94℃ for 30 s, 55℃ for 30 s, and 72℃ for 30 s, and in the last step 72℃ for 7 min for final extension was performed. To ensure the accuracy of the PCR reaction, 3 μl of PCR product stained using GelRed (Fermentase, USA) was subjected to electrophoresis on 1.5% agarose gel prepared in 1X TBE buffer at 80 V for 40 min. Finally, the amplified segment was transferred to Macrogen Company (South Korea) and sequencing was performed. Finally, data from Macrogen compared with those in GenBank using the BLAST program and strains with more than 97% similarity to the reference were allocated to the same species (Vasiee, Mortazavi, et al., 2018).

### 2.2 Probiotic analysis of Lactobacillus isolates

#### 2.2.1 Resistance to low pH

The isolates were grown separately for 18–24 hr in 5 ml MRS broth at 37℃ under anaerobic conditions. The grown bacteria were centrifuged at 6,000 × *g* for 10 min at 4℃. Afterward, the pellets were rinsed 2 times and re‐suspended in the PBS buffer (Sigma‐Aldrich) and the pH adjusted to 2 and 3. After this procedure, they were incubated for 0, 1, 2, and 3 hr at 37℃. Finally, the number of survival bacteria was counted by serial dilution method on the MRS agar plates. The number of living lactobcilli was reported as log colony‐forming unit (CFU)/mL. In all the probiotic tests, *Lactobacillus rhamnosus* GG ATCC 53103 was used as a standard strain for qualitative comparison (Topçu et al., 2020).

#### 2.2.2 Resistance to simulated gastric and intestinal fluid

An overnight culture of 30 ml of MRS broth containing each strain was centrifuged at 8,000 × *g* for 5 min at 4℃. The supernatant removed and the collected cells were washed twice with 10 ml of 50 mM PBS (pH=6.5) and after that, re‐suspended in 3 ml PBS buffer. One milliliter of each strains containing 9 log CFU/ml of bacteria was added to 9 ml of simulated gastric fluid (NaCl 125 mM, KCl 7 mM, NaHCO3 45 mM, pepsin (3 g/L) (Sigma‐Aldrich), pH=2.5). The prepared gastric fluid was incubated at 37℃ for 3 hr. Afterward, the suspension was subjected to centrifuge at 3,800 rpm for 10 min, the supernatant was removed and the pellets were washed with PBS. The pellet re‐suspended in simulated intestinal fluid containing pancreatin 0.1% w/v (Sigma‐Aldrich), bile salt 0.15% w/v, pH=8.0 and incubated for 3 hr at 37℃. After incubation, the numbers of survival bacteria were counted and reported as log CFU/ml (Grimoud et al., 2010; Shukla & Goyal, 2014).

#### 2.2.3 Resistance to bile salt

The ability of each isolates to grow in the media containing of bile salt (Oxgall; Himedia, Mumbai, India) was investigated based on the method suggested by Vasiee, Alizadeh Behbahani, et al. (2018) with some modification. All the strains were inoculated in the MRS broth containing 0, 0.3, 0.5, and 1.0% of bile salt. The cultures were incubated at 37℃ for 18 hr. Afterward, the growth of strains was recorded at 600 nm (Vasiee, Alizadeh Behbahani, et al., 2018).

#### 2.2.4 Cell surface hydrophobicity assay

The potential of the strains to adhere to nonpolar solvent as a measure of their hydrophobicity was determined according to Jena et al. (2013). Strains were centrifuged 6,000 × *g* for 15 min, the pellet was washed twice in with PBS. After that, the strains were re‐suspended in PBS buffer till the optical density at 600 nm reach 0.6–0.7 (OD_0_). Three milliliters of each re‐suspended strain were added to 1 ml of n‐ hexadecane (Merck, Germany). After 15 min of incubation at room temperature, each tube was vortexed for 3 min. Then, it was kept at room temperature for 30 min. Afterward, the absorbance of aqueous phase was measured (OD) (Jena et al., 2013).

$$\text{Hydrophobicity}\%=\left[\frac{{\text{OD}}_{0}‐\text{OD}}{{\text{OD}}_{0}}\right]\times 100.$$

#### 2.2.5 Auto‐aggregation and coaggregation

Auto‐aggregation abilities of the strains were determined according to the method described by Kos et al. (2003) with brief modifications. Overnight culture of Lactobacillus strains was centrifuged at 6,000 × *g* for 15 min at 4℃ and the supernatant was removed. The pellet was washed by PBS and re‐suspended in the same buffer to reach the number of bacteria to 8 log CFU/ml. The suspension of each strain was incubated at 37℃ for different time periods (0, 2, 4, 6, 12, and 24 hr). Auto‐aggregation was determined in percentage according to the following equation (Kos et al., 2003):

$$\text{Auto - aggregation \textbackslash{}\% =}\;\left[\frac{A_{0}‐A_{1}}{A_{1}}\right]\times 100.$$

where *A_t_* and *A*_0_ represent the absorbance at different times and the absorbance *t* = 0, respectively.

For coaggregation, Lactobacillus strains as well as pathogenic bacteria (including *Escherichia coli* ATCC 25922, *Pseudomonas aeruginosa* PTCC 1707, *Salmonella enterica* serovar Typhimurium ATCC 14028, and *Staphylococcus aureus* ATCC 25923.) were cultured overnight and after that, suspensions were prepared as described previously and OD_600nm_ was adjusted to 0.2–0.3. Afterward, 2 ml of lactobacilli strain was mixed with 2 ml of the various suspensions of each pathogens for 10 s and incubated at 37℃ for 5 hr. Coaggregation was calculated according to the following equation:

$$\text{Co - ggregation}\%=\left[1‐\frac{A_{m}}{\frac{A_{l}+A_{p}}{2}}\right]\times 100.$$

Where *A_m_* shows the absorbance of the mixture suspension. *A*_l_ and *A_p_* represent the absorbance of Lactobacillus strains and pathogenic bacteria, respectively (Falah et al., 2019).

#### 2.2.6 Adhesion capacity

The Caco‐2 cells were cultured in Dulbecco's Modified Eagle's (DMEM, Sigma) broth supplemented with 10% fetal bovine serum (Sigma) and 1% penicillin‐streptomycin (Sigma) in the CO_2_ incubator for cell culture until it became 80% confluency. Afterward, Caco‐2 cells were detached and the cells were cultured in 6‐well plates with 125.000 cells/well concentration. 6‐wells plates were placed at 37℃ with medium change every 2 day. After that, the bacterial suspension of each strain (in the DMEM media) with 8 log CFU/ml was prepared, added to the wells, and incubated at 37℃ for 1 hr. The wells were washed with cold and sterile PBS to remove the nonbound bacteria. The Caco‐2 cells and Lactobacillus strains were detached using 100 μl Triton‐X100 (Sigma). After 10 min incubation at 37℃, MRS broth was added, pipetting was done and finally, bacterial culture was performed (Hojjati et al., 2020). The percentage of bacterial adhesion was calculated as:

$$\text{Adhesion}\%=\left[\frac{\text{adhered bacteria}}{\text{Initial number of bacteria}}\right]\times 100.$$

### 2.3 Technological properties

#### 2.3.1 Acidifying ability

The Lactobacillus strains were inoculated into MRS broth for reactivating of isolates and incubated at 37℃ for 18 hr. The bacterial suspension subjected to centrifuge process at 6,000 × *g* for 15 min. The pellets were washed with sterile and cold PBS and after that, re‐suspended with 10 ml of sterile skimmed milk (Merck). The new suspension incubated at 37℃, and ∆pH was recorded with a pH‐meter (Metrohm, Switzerland) after 6 and 24 hr of incubation (Nezhad et al., 2020).

ΔpH = pH(at time 6 and 24 h) - pH(zero time).

#### 2.3.2 Proteolytic activity

The proteolytic activity of Lactobacillus isolates was assessed on skim milk agar. Fifty microliters of each strain supernatant were placed on the center of the plate, in a well 6 mm wide. Afterward, plates kept for 48 hr at 37℃. The clear halo indicates the proteolytic activity of the strains which was recorded as the diameter of the clear zone (mm) (Nespolo & Brandelli, 2010).

#### 2.3.3 Lipolytic activity

To qualitatively study lipolytic activity of Lactobacillus strains, Cream fat agar plates (Merck, Germany) containing milk cream (1% (v/v)) were used from which bacteria streaked. Then, the plates were incubated at 37℃ for 48–72 hr. Positive lipolytic activity was recorded when colonies surrounded by a clear ring (Nieto‐Arribas et al., 2009).

#### 2.3.4 Autolytic activity

All Lactobacillus strains were cultured separately on MRS broth till their OD_600nm_ reached 0.7–0.8. After that, they were centrifuged at 6,000 × *g* for 15 min at 4℃. Pellets were washed with 20 mM sodium phosphate monobasic buffer (pH 6.8) and re‐suspended in the same buffer. The pellets were incubated at room temperature for 4 hr and changes in OD_600nm_ were recorded.

$$\text{Autolytic activity}:100‐(A_{1}/A_{2})\times 100.$$

*A*₁ and *A*₂ are lowest and the highest degrees of OD_600nm_.

### 2.4 Antimicrobial activity

Antimicrobial activity of Lactobacillus isolates was assessed based on the method introduced by Jena et al. (2013). Four pathogenic strains were used as an indicator to investigate the antimicrobial activity of the Lactobacillus strains. They included *E*. *coli* ATCC, *P*. *aeruginosa*, *S. enterica* serovar Typhimurium, and *S*. *aureus*. Lactobacillus strains were cultured on MRS broth at 37℃ for 18 hr, centrifuged at 6,000 × *g* for 15 min, and finally, the supernatant was filtrated by filtration sterilization. After that, the filtrates were made neutral with 5 N NaOH (Merck, Germany) till the pH reached 6.5. Overnight culture of pathogens (10^8^ CFU/ml) was cultured on the MRS agar (Merck, Germany) which 6 mm diameter wells were punctured in each plate. One hundred µL of prepared supernatants was poured into wells and kept at 37℃ for 24 hr and finally, the inhibition zone was measured.

### 2.5 Safety evaluation

#### 2.5.1 Antibiotic susceptibility

The antibiotic susceptibilities of Lactobacillus strains were assessed with tetracycline, kanamycin and chloramphenicol (30 mg per disc), and erythromycin (15 mg per disc). The strains were streaked with concentration of 8 log CFU/ml in MRS agar plates and placed the antibiotic discs on the media surface. The plates were incubated for 24–48 hr at 37℃ and afterward, the inhibition zone surrounding the discs was recorded. Based on Clinical and Laboratory Standards Institute tables (CLSI, 2015), the isolates considered in three categories: resistant (R), intermediate (I), and sensitive (S) (Fortina et al., 2008).

#### 2.5.2 Hemolytic activity

Hemolytic activity of Lactobacillus bacteria was investigated by culturing the isolates on Tryptic Soy Agar (Merck, Germany) with sheep blood (7% (v/v)). The plates were kept for 24 hr at 37℃, and any changes on the plates were reported. γ‐hemolysis or α‐hemolysis was considered nonhemolytic; while β‐hemolysis was considered as hemolytic (Casarotti et al., 2017).

#### 2.5.3 Biogenic amine (BA) production

The ability of Lactobacillus strains to produce BA by decarboxylation of amino acids was tested on a media designed by Yousif et al. (2005) which contained the precursor amino acids including L‐histidine monohydrochloride, tyrosine di‐sodium salt, L‐ornithine monohydrochloride, and L‐lysine monohydrochloride (Acros & Bio basic). First, Lactobacillus spp. were subcultured twice in MRS broth containing 0.1% of each precursor amino acid and 0.005% pyridoxal‐5‐phosphate. After that, strains were spotted on the MRS agars with and without amino acids which containing 0.06% bromocresol purple (Sigma). After 2–5 days incubation, purple color obtained in surrounding colonies was considered as a positive (Yousif et al., 2005).

## 3 RESULTS AND DISCUSSION

### 3.1 Isolation and identification of Lactobacillus strains

After grouping the bacteria isolated from the local cheese of Ahvaz province, based on carbohydrate fermentation profile, 4 different groups were created for bacilli‐shaped bacteria. 1–2 bacteria were selected from each group, DNA extraction and PCR amplification based on 16S rDNA gene were performed and the results of sequencing are shown in Table 1.

**TABLE 1:** Lactobacillus isolates identification

Strain	Closest relative	Identity (%)	GenBank accession no.
B2	*Lactobacillus brevis*	98	MN749954.1
B3	*L. brevis*	97	MG646884.1
B14	*Lactobacillus acidophilus*	100	HM162411.1
B15	*L. acidophilus*	97	KC150145.1
B17	*L. acidophilus*	99	MN173898.1
B20	*Lactobacillus plantarum*	99	MN049548.1
B22	*Lactobacillus casei*	97	NR041893.1

LAB are found in local cheeses and play a major role in ripening through biochemical reactions. Since adjunct cultures play an important role in creating flavor in cheese, identification of this group of bacteria for industrial applications is very important (Guarrasi et al., 2017). PCR is one of the most powerful methods in identifying different types of bacteria based on the replication of genetically conserved regions. The advantages of this method include high speed, accuracy, and precision, which do not exist in the culture‐based methods (Boldura & Popescu, 2016). Van Hoorde et al. (2008) identified and grouped of LAB associated with the production of two artisanal raw milk cheeses. The results of sequence analysis showed the Lactobacillus spp. belonged to *L. plantarum*, *L. brevis*, *L*. *rhamnosus*, *L. paracasei*, and *L perolens*, *L. curvatus* (Van Hoorde et al., 2008). Azizi et al. (2017) identified Lactobacillus spp. from Iranian local cheese and reported that Lactobacillus spp. was belonged to *L. plantarum*, *L. casei*, *L. brevis,* and *L. buchneri* (Azizi et al., 2017). Singh and Singh (2014) reported several Lactobacilli spp. have been isolated from ripened cheddar cheese including *L. rhamnosus*, *L. paracasei*, *L. plantarum*, *L. brevis*, and *L. curvatus* (Singh & Singh, 2014).

### 3.2 Beneficial properties of Lactobacilli isolates

#### 3.2.1 Tolerance to the GIT conditions

Survival under low pH, bile salts, intestinal, and gastric juices is known as a critical feature for the selection of potential probiotic candidates. In fact, the first condition for a probiotic bacterium is to be able to reach its target point in the digestive system, and for this purpose, it must be able to survive in difficult conditions of the gastrointestinal tract (Todorov et al., 2012). The resistances of the selected Lactobacillus strains to acidic condition, gastric and intestine fluid are presented in Table 1.

According to the obtained results, after 3 hr incubation at pH of 2, 3–6 log units decrease depending on the strain in the number of bacteria was observed. While this decrease was not observed under pH 3, except for *L. casei* strain, which decreased about 1.5 log unit after 3 hr incubation (Table 2). The highest survival rate in acidic conditions was related to *L. acidophilus* (B14), which was even higher than standard strain. All Lactobacillus spp. (except *L. casei*) can be considered as probiotic candidates in terms of this feature. Various factors can increase the resistance of probiotic bacteria to acidic conditions in the stomach, including the food matrix that acts as a carrier of probiotic bacteria, which can increase the pH and resulting longer bacteria survive. Probiotic bacteria can also adapt their survival to acidic conditions through F0F1‐ATPase mechanism. Some other factors can affect pH resistance including the composition of growth medium, composition of the cytoplasmatic membrane, nutritional compounds, and the type of bacteria (Feyhl‐Buska et al., 2016). Azat et al. (2016) studied the pH resistance of LAB isolated from traditionally fermented Xinjiang cheese and reported that all tested strains showed resistance to low pH because after incubation at pH 3 for 3 hr the viable counts of all strains were found to be >10^6^ CFU/ml. Usman and Hosono (1999) indicated that for selected probiotic bacteria, the survival rate at pH 3 is considered as optimal acid tolerance. Six strains of Lactobacillus spp. from this study were capable to resist pH 3.0 and therefore they can be considered as acid tolerant (Usman & Hosono, 1999).

**TABLE 2:** The resistance of Lactobacillus spp. to pH 2 and 3, simulated gastric and intestinal fluid (log CFU/ml)

Strains	pH=2				pH=3				Simulated GIT juices
	0 hr	1 hr	2 hr	3 hr	0 hr	1 hr	2 hr	3 hr	
*Lactobacillus brevis* (B2)	9.12 ± 0.23	8.35 ± 0.16	7.65 ± 0.27	5.81 ± 0.20	9.36 ± 0.28	9.25 ± 0.14	9.12 ± 0.15	9.52 ± 0.18	7.20 ± 0.27
*L. brevis* (B3)	9.05 ± 0.13	6.56 ± 0.11	7.13 ± 0.22	4.20 ± 0.26	9.15 ± 0.30	9.45 ± 0.20	9.63 ± 0.19	9.31 ± 0.15	6.10 ± 0.20
*L. acidophilus* (B14)	9.02 ± 0.14	7.56 ± 0.18	7.95 ± 0.25	6.26 ± 0.19	9.05 ± 0.24	9.10 ± 0.13	9.36 ± 0.21	9.48 ± 0.11	7.40 ± 0.25
*L. acidophilus* (B15)	9.22 ± 0.19	6.23 ± 0.17	6.10 ± 0.21	4.65 ± 0.22	9.20 ± 0.26	9.22 ± 0.15	9.12 ± 0.18	9.16 ± 0.19	7.15 ± 0.29
*L. acidophilus* (B17)	9.16 ± 0.22	8.05 ± 0.25	5.96 ± 0.19	4.23 ± 0.18	9.45 ± 0.27	9.56 ± 0.11	9.32 ± 0.17	9.25 ± 0.17	7.05 ± 0.19
*L. plantarum* (B20)	9.00 ± 0.20	5.98 ± 0.20	5.36 ± 0.19	4.80 ± 0.17	9.18 ± 0.18	9.05 ± 0.26	9.12 ± 0.27	8.95 ± 0.25	6.80 ± 0.13
*L. casei* (B22)	9.10 ± 0.15	6.15 ± 0.24	5.25 ± 0.17	3.56 ± 0.23	9.05 ± 0.20	8.84 ± 0.18	8.15 ± 0.16	7.59 ± 0.20	5.90 ± 0.18
*L. rhamnosus* GG	9.06 ± 0.17	8.25 ± 0.0.23	7.58 ± 0.11	6.12 ± 0.17	9.14 ± 0.14	9.58 ± 0.13	9.50 ± 0.25	9.35 ± 0.10	7.38 ± 0.15

Human saliva with high lysozyme concentration is the first obstacle that need to be overcome. Rest of obstacles are the upper gastrointestinal tract, which contains the stomach with the low pH and the small intestine with bile salts and digestive enzymes which the probiotic strain should pass and be alive with no significant reduction in number. In this study, the initial number of 9 log CFU/ml inoculations was used. After exposure to the simulated gastric and intestinal conditions, changes in their number were observed depending on the type of strain. Except *L. casei* B22, *L. brevis* B3, and *L. plantarum* B20, all other strains could be considered as resistant to this condition. Compared to the standard strain, *L. acidophilus* (B14) had the highest resistance to simulated intestinal and gastric conditions. Ashraf and Smith (2016) investigated the resistance of commercial LAB to harsh condition of GIT. They reported that all tested LAB showed resistance during simulated gastric transit for 3 hr, at pH 3. Also, *L. rhamnosus* G5435, *L. reuteri*, *L. delbrueckii* subsp. *bulgaricus* 11,842, *L. acidophilus* 388, *Bifidobacterium lactis* BB12, *Streptococcus thermophilus* 1,342, and *S. thermophilus* M5 were found to be tolerant to gastric and small intestinal transit (Ashraf & Smith, 2016). Feng et al. (2017) evaluated the resistance of some LAB isolated from the intestinal mucosa of healthy piglets and reported that 2 *E. faecium* and one *L. plantarum* exhibited good simulated upper gastrointestinal tract tolerance (Feng et al., 2017).

The small intestine and the clone in human body contain high amounts of bile salts which can kill probiotic bacteria; therefore, it is necessary to investigate the bile salt resistance of isolates (Kumar et al., 2012). In this study, all bacteria were able to survive and grow at different concentrations of bile salts. However, the rate of growth depends on the concentration of bile salts. Lactobacillus spp. gave percentages of growth ranging from 86% to 98% at the concentration of 0.3% bile salt. Lactobacillus strains showed good resistance and growth ranging from 84%–100% at 0.5% and 74 to 99% at 1% bile salt. These results are similar to other studies which have showed that LAB could remain survival at high bile degrees (Abushelaibi et al., 2017; Boricha et al., 2019; Mulaw et al., 2019; Olatunde et al., 2018; Shehata et al., 2016).

#### 3.2.2 Cell surface hydrophobicity

**FIGURE 1:**
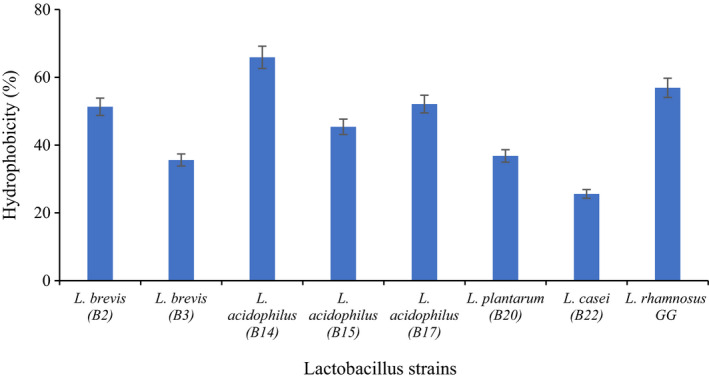
Hydrophobicity potential of Lactobacillus spp

Hydrophobicity and auto‐aggregation categorized as phenotypic properties which directly attributed to the strain adhesion ability. Different compounds including n‐hexadecane, xylene, ethyl acetate, and chloroform can be used to distinguish the hydrophobicity. Hydrophobicity is a interaction result between the bacteria and the host cell and can be calculated in vitro by mixing bacterial strains suspension with one of the nonpolar compounds mentioned through reading aqueous solution adsorption (OD_600nm_) before and after mixing. The results of hydrophobicity of Lactobacillus spp. are shown in Figure 1. The highest hydrophobicity is related to *L*. *acidophilus* B14 (65.9%), and the lowest is related to *L. casei* B22 (25.6%).

Guan et al. (2020) assessed the hydrophobicity properties of 6 LAB isolated from the longevous population of China and reported this feature varies from 14.8% to 57.3%. Differences in hydrophobicity rates might be mainly caused by chemically and structurally heterogeneous bacterial surface, such as cell wall intercalated proteins, hydrophobic amino acids, cytoplasmic membrane protein, polysaccharides, surface array proteins, and lipids. Moreover, there are also some other factors that can affect the degree of hydrophobicity including cell growth phase, environmental factors, and degree of pleomorphism (Guan et al., 2020).

#### 3.2.3 Auto‐aggregation and co‐aggregation ability

**FIGURE 2:**
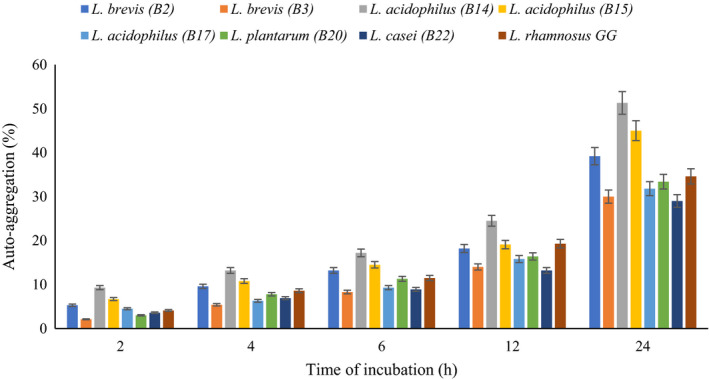
Auto‐aggregation percentage of lactobacilli spp. as measured at 2, 4, 6, 12, and 24 hr of incubation at 37℃

**FIGURE 3:**
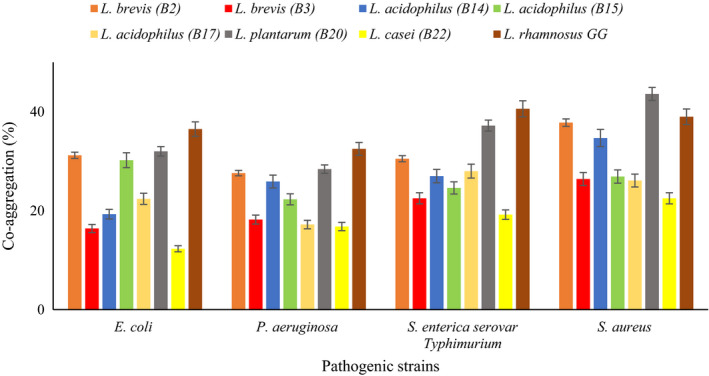
Co‐aggregation percentages of lactobacilli spp. with pathogens measured after 24 hr of incubation at 37℃

Auto‐aggregation is directly related to the adhesion potential of probiotic bacteria while co‐aggregation associated close interaction with pathogens. The results of auto‐aggregation and co‐aggregation values of Lactobacillus spp. are shown in Figures 2 and 3, respectively. Auto‐aggregation increased when the incubation time increased. Among the isolates, *L. acidophilus* B14 showed the highest auto‐aggregation degree (51.3%) after 24 hr of incubation, followed by *L. acidophilus* B15 (45.0%) and *L. brevis* B2 (39.2%).

The bacterial auto‐aggregation and cell surface hydrophobicity ability are directly correlated. The strains attach to cell monolayers of human intestine if they have strong hydrophobicity and auto‐aggregate (Patel et al., 2011).

All Lactobacillus strains were able to co‐aggregate with pathogen bacteria (Figure 3). The co‐aggregation degree of standard strain with all pathogens (except *S*. *aureus*) was higher than all tested isolates. *L. plantarum* B20 and *L. brevis* B2 had higher degree of co‐aggregation with pathogens. *L. plantarum* B20 had a high co‐aggregation with *S. aureus* (43.6%) and a lesser one with *E. coli* (32.0%). Likewise, *L. brevis* B2 showed strong co‐aggregation with *S. aureus* (37.8%) but was weaker with *P. aeruginosa* (27.6%). *L. casei* B22 showed weakest co‐aggregation with *E. coli* (12.3%). The auto‐aggregation property helps bacteria to attach to the intestinal cells and mucosal surfaces while the co‐aggregation may enable them to form a barrier that prevents colonization and biofilm formation of pathogenic bacteria (Cozzolino et al., 2020). Jena et al. (2013) reported auto‐aggregation of Lactobacillus strains isolated from rat fecal microbiota were between 33.2% and 47.2% while co‐aggregation of them with pathogenic strains were ranged between 11.89% and 38.22%, and the highest rate of co‐aggregation was observed with *S. aureus*. Tuo et al. (2013) studied aggregation and adhesion properties of some Lactobacillus strains. They treated the strains with guanidine HCl, so the auto‐aggregating and adhering abilities of some *Lactobacillus* strains decreased. These results indicate that surface‐bound proteins and other macromolecules played an important role in the auto‐aggregating abilities and adhering (Tuo et al., 2013). Grigoryan et al. (2018) evaluated the auto‐aggregation of some lactobacilli and reported *L. paracasei* CCMA 0,505 (52.66%) and *L. paracasei* CCMA 0,504 (45.36%) showed the highest auto‐aggregation degrees (Grigoryan et al., 2018).

#### 3.2.4 Adhesion capacity

In order for probiotic bacteria to have a positive effect, they must be able to reach intestinal cells in sufficient quantities and alive. Analysis in vivo adhesion ability of LAB is difficult, therefore, probiotic bacterial adhesion often examined using in vitro model cells such as HT‐29, T84, Caco‐2, and mucous‐secreting HT‐29MTX which derived from colon adenocarcinoma cells and are morphologically very similar to human intestinal cells.

**FIGURE 4:**
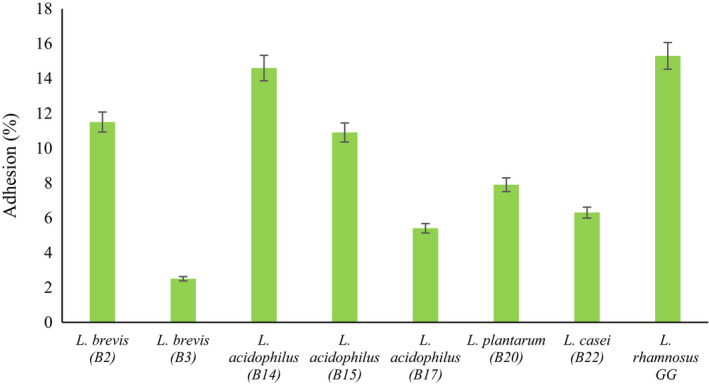
Adhesion capacity of Lactobacillus strains to the Caco‐2 cell line

All the isolates were able to adhere to Caco‐2 cells with different degrees. The adhered percentage of bacteria varied from 2.5% to 14.6% which was less than the standard bacterial adhesion (Figure 4). The highest amount belonged to *L*. *acidophilus* B14 and the lowest amount related to *L*. *brevis* B3. Few studies have reported that exopolysaccharides and lipoteichoic acids on the cell walls of Lactobacillus strains contain adhesive molecules which can improve their adhesion ability. Lactobacillus spp. have some proteins that can bound weakly (by noncovalent interaction) to surface components of intestinal cells (Bergonzelli et al., 2006; Tallon et al., 2003). The adhesion ability of probiotic bacteria is initially started with nonspecific physical interactions between two surfaces, which then cause specific interactions between adherence components and complementary receptors. Auto‐aggregation and hydrophobicity activity play important roles for initial contact between the isolate and cell (Guan et al., 2020). García‐Ruiz et al. (2014) investigated the probiotic properties of LAB isolated from wine and reported adhesion degrees of LAB strains varied between 0.37% and 12.2%, depending on the strain (García‐Ruiz et al., 2014). Falah et al. (2019) analyzed the adhesion level of *L. fermentum* isolated from Tarkhineh, an Iranian cereal‐dairy product and reported the adhesion capacity of this strain was 8.5%.

### 3.3 Technological properties

#### 3.3.1 Acidifying activity

**FIGURE 5:**
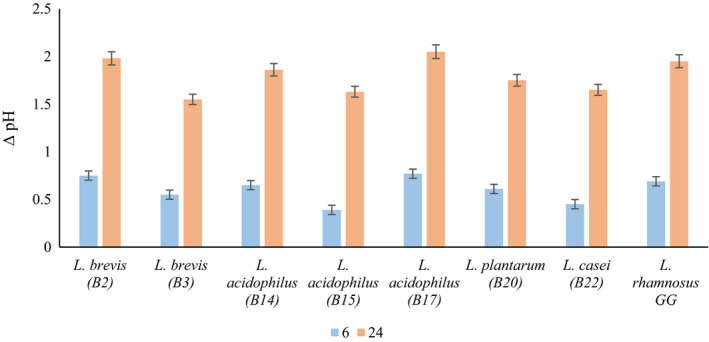
Changes of pH in Reconstituted Skim Milk (RSM) medium after 6 and 24 hr

After 6 and 24 hr of incubation, reduced pH changes by the Lactobacillus spp. record between 0.39–0.75 and 1.55–2.05, respectively (Figure 5).

According to the classification of Nieto‐Arribas et al. (2009), isolates are divided into three groups according to produce acidic compound after 24 hr of incubation: 1) those with high acid capacity with a pH decrease more than 2 units; 2) those with medium acidity capacity with the ability to reduce the pH in the range of 1.5–2 units; and 3) those with low acidity capacity with the ability to reduce the pH in less than 1.5 units (Nieto‐Arribas et al., 2009). According to this classification, all Lactobacillus strains except *L. acidophilus* B17 had moderate acidity capacity and reduced the pH between 1.5 and 2 units. One of the important characteristics of LAB that are used as a starter, adjunct culture, or supplement in various fermented food products is their potential to create acidic conditions. The production of acid, after which the pH decreases, causes a special flavor in the product and can also kill pathogenic and spoilage bacteria, and the safety and shelf life of the product significantly increase. It should be noted that in fermented products, a slow decrease in pH is not desirable and can cause changes in the microbiological and textural of the product (Tilocca et al., 2020). Ma et al. (2012) reported Lactobacillus spp. did not reach the pH below 5.3 after 6 hr and classified them as low acid production (Ma et al., 2012). Ebadi Nezhad et al. (2020) reported that Lactobacillus spp. reduced pH between 0.67 and 2.06 units after 24 hr which indicates that these strains have low potential to acidify the environment.

#### 3.3.2 Proteolytic activity

**FIGURE 6:**
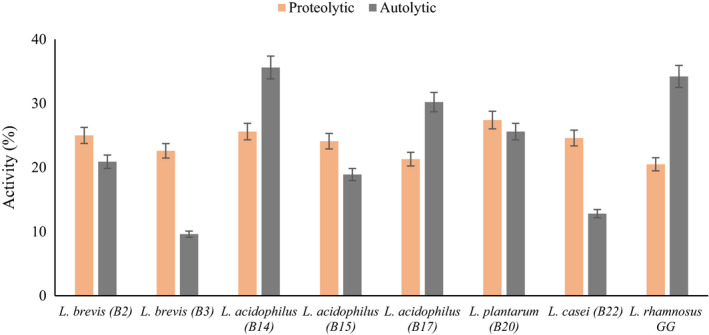
Proteolytic and autolytic activity (%) of lactobacillus spp

Casein degradation in connection with the proteolytic and peptidolytic activity of microorganisms plays an improving role in flavor development, acid production, and cheese ripening. Few peptides help to create the desired flavor in the product. In addition to the native proteolytic enzymes of milk and renin enzymes involved in protein coagulation, protease, and intracellular peptidases are released into the curd after lysis of the LAB cell wall, which plays an important role in casein hydrolysis during cheese production. The highest proteolytic activity belonged to *L. plantarum* B20 followed by *L. acidophilus* B14 (Figure 6). Although LAB are usually considered weak in proteolytic activity, application of strains with high proteolytic potential for the production of hard cheeses is necessary. For ripening the semihard cheeses, low proteolytic activity LAB could be used (Ebadi Nezhad et al., 2020). Sasaki et al. (1995) compared the proteolytic activities in various lactobacilli strains and reported that the proteolytic activity of the LAB is different within each species.

#### 3.3.3 Lipolytic activity

In this study, Lactobacillus bacteria showed no lipolytic activity. Our results are consistent with the results reported by Pérez et al. (2003) who examined the technological properties of Tenerife cheese (Pérez et al., 2003), as well as the studies of Nieto‐Arribas et al. (2010) who studied and investigated Manchego cheese (Nieto‐Arribas et al., 2010) and found that Lactobacillus bacteria did not show lipolytic activity after culturing in the tributyrin agar medium. The lack of lipolytic activity seen in our study could suggest Lactobacillus spp. as a better candidate for the dairy industry. Poor lipolysis of milk fat causes flavor in the final product but does not improve further and also does not cause rancidity.

#### 3.3.4 Autolytic activity

Autolytic activity is one of the interesting properties of LAB that leads to the release of intracellular lipase and protease enzymes that are effective in improving sensory and textural characteristics. According to the grouping proposed by Ayad et al. (2004), 42.8% of isolates in our study showed good autolytic activity (activity between 25% and 65%), 28.6% had relatively good autolytic activity (activity between 15% and 24%), and 28.6% of isolates showed low autolytic activity (below 14%) (Figure 6). The highest autolytic activity belonged to *L. acidophilus* B14 and the lowest belonged to *L. brevis* B3. Ebadi Nezhad et al. (2020) investigated the autolytic properties of strains isolated from Motal cheese. The results of their study showed that the autolytic activity of 16 lactobacilli isolates was recorded between 6.28% and 38.28% which 25% of the isolates had good autolytic activity; 50% of the isolates had relatively good autolytic activity and the rest of the isolates with autolytic activity below 14%, were considered weak. It is known that some LAB can release some hydrolase through the bacterial cell wall and rupture the peptidoglycan; this process can be influenced by various factors such as carbon source, temperature, osmotic concentration, and pH. N‐acetylmuramidase has a critical function in the autolysis of *L*. *bulgaricus* and is known as one of the major causes of cell wall destruction (Pang et al., 2014).

### 3.4 Antimicrobial activity

**FIGURE 7:**
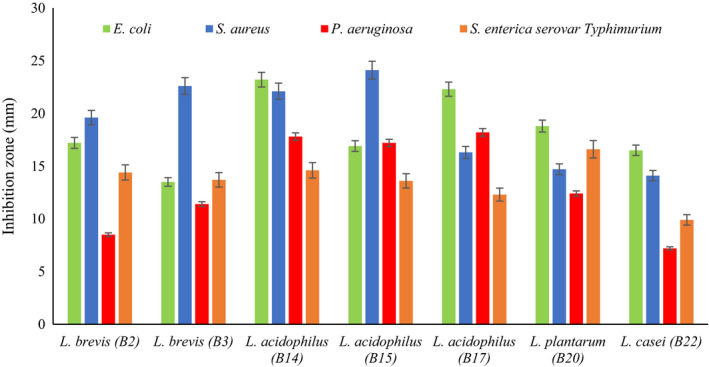
Antimicrobial activities of neutral pH supernatants of lactobacilli spp. against pathogenic strains

The results of antibacterial activity of the Lactobacillus spp. are shown in Figure 7. All 7 strains (in the form of neutral cell‐free supernatant) inhibited the growth of indicator bacteria; however, the degree of inhibition was different between the strains. In particular, strain *L*. *acidophilus* B14 showed the strongest inhibitory activity against the pathogens and inhibition zone ranged from 14.6 to 23.2 mm but strain *L. casei* B22 had the lowest inhibitory activity against the pathogens with inhibition zone ranged from 7.2 to 16.5 mm.

The antimicrobial activity of LAB is important because these bacteria must be able to inhibit and kill the growth of potentially pathogenic bacteria in fermented products as well as in the intestinal environments. The antibacterial effect of these bacteria can be attributed to metabolites such as organic acids, hydrogen peroxide, diacetyl, ethanol, phenols, and protein compounds that they produce during growth. The most common antimicrobial compounds reported to be produced by probiotic bacteria include bacteriocins, hydrogen peroxide, and organic acids (especially lactic and acetic acids). These metabolites, together with help of competitive exclusion mechanism, in which probiotic compete with harmful bacteria for adhesive receptors and nutrients, could destroy and prevent colonization of pathogens in the body.

### 3.5 Safety attributes

All strains showed no hemolysis activity. Also, one of the 7 strains produced detectable quantities of tyramine (*L. brevis* B3). Production of other BA from the precursors could not be detected. BA formed mainly by amination and transamination of aldehydes and ketones or decarboxylation of amino acids and usually found in foods that are free of proteins and amino acids which subjected biochemical or microbial processes such as fermentation. BA may be present in fish‐related foods, fermented sausages, cheese, and some other fermented foods. The absence of histamine and tyramine is usually further investigated for its toxic and allergic effects. Although the production of BA has been reported by some LAB strains such as Enterococcus and Lactobacillus, their absence in food is an important principle (Yousif et al., 2005).

Another characteristic of probiotic bacteria that should be examined is their resistance to common antibiotics which determine their safety for food consumption. The antibiotic‐resistant properties of Lactobacillus strains to four clinical antibiotics were evaluated according to the CLSI and the data are presented in Table 3. According to guidelines of CLSI, none of the isolates were resistant to chloramphenicol and erythromycin. Also, 57% and 28% of isolates were resistant to kanamycin and tetracycline, respectively.

**TABLE 3:** Antibiotic susceptibility of Lactobacillus strains

Strain	Erythromycin			Kanamycin			Tetracycline			Chloramphenicol		
	S x ≥ 23	I 14–22	R x ≤ 13	S x ≥ 18	I 14–17	R x ≤ 13	S x ≥ 15	I 12–14	R x ≤ 11	S x ≥ 18	I 13–17	R x ≤ 12
*Lactobacillus brevis* (B2)	23.75 ± 0.23 (S)			14.12 ± 0.25 (I)			13.20 ± 0.42 (I)			19.85 ± 0.47 (S)		
*L. brevis* (B3)	17.75 ± 0.56 (I)			11.30 ± 0.56 (R)			10.56 ± 0.13 (R)			18.65 ± 0.25 (S)		
*L. acidophilus* (B14)	23.35 ± 0.78 (S)			15.74 ± 0.48 (I)			15.85 ± 0.36 (S)			21.45 ± 0.65 (S)		
*L. acidophilus* (B15)	19.78 ± 0.35 (I)			12.80 ± 0.38 (R)			10.85 ± 0.75 (R)			16.75 ± 0.38 (I)		
*L. acidophilus* (B17)	24.20 ± 0.65 (S)			14.50 ± 0.24 (I)			15.35 ± 0.56 (S)			22.14 ± 0.36 (S)		
*L. plantarum* (B20)	25.31 ± 0.47 (S)			12.80 ± 0.41 (R)			12.75 ± 0.30 (I)			20.50±0.036 (S)		
*L. casei* (B22)	18.30 ± 0.33 (I)			13.40 ± 0.65 (R)			13.52 ± 0.47 (I)			19.35 ± 0.47 (S)		

Bacterial susceptibility to antibiotics varies among genus and species belonging to the same family and should be evaluated for each strain. For some strains, resistance to antibiotics is intrinsic and cannot be transmitted to other bacteria. However for others, it is possible to transfer the antibiotic resistance gene, but there are concerns about the commercially use of these bacteria, as it is possible to transfer this gene to pathogenic bacteria and cause them resistant (Vasiee, Falah, Behbahani, et al., 2020). Jena et al. (2013) reported that all the lactobacillus strains were susceptible to all studied antibiotics, except vancomycin; and recognized these isolates as intrinsically resistant to vancomycin. Agostini et al. (2018) reported that all lactobacilli studied were sensitive to chloramphenicol, erythromycin, ampicillin, and tetracycline. They also showed that most of the studied strains are resistant to vancomycin, which is in line with our study results (Agostini et al., 2018).

## 4 CONCLUSION

In summary, seven lactobacillus spp. isolated from Iranian local raw milk cheese from Ahvaz province, have in vitro investigations that make them potential candidates for probiotic and technological applications. The results showed that these strains have good probiotic and technological potential. The results of safety aspects also showed that these strains can be used for human consumption. Hence further, in vivo trials are needed to investigate their performance in real‐life situations.

## CONFLICT OF INTEREST

The authors have declared no conflict of interest.

## AUTHOR CONTRIBUTION

**Hasan Barzegar:** Data curation (equal); Formal analysis (equal); Project administration (equal); Supervision (equal); Validation (equal); Writing‐review & editing (equal). **Behrooz Alizadeh Behbahani:** Formal analysis (equal); Methodology (equal); Software (equal); Validation (equal); Writing‐original draft (equal). **Fereshteh Falah:** Investigation (equal); Methodology (equal); Resources (equal); Software (equal); Writing‐original draft (equal).

## ETHICAL APPROVAL

This article does not contain any studies with human or animal subjects.
